# Key Fundamentals and Examples of Sensors for Human Health: Wearable, Non-Continuous, and Non-Contact Monitoring Devices

**DOI:** 10.3390/s25020556

**Published:** 2025-01-19

**Authors:** Sara Guarducci, Sara Jayousi, Stefano Caputo, Lorenzo Mucchi

**Affiliations:** 1 Department of Information Engineering, University of Florence, 50139 Florence, Italy; sara.guarducci@unifi.it (S.G.); stefano.caputo@unifi.it (S.C.); lorenzo.mucchi@unifi.it (L.M.); 2 PIN Foundation—Prato Campus, University of Florence, 59100 Prato, Italy

**Keywords:** information and communication technologies, internet of things, sensors, personalized healthcare, health monitoring

## Abstract

The increasing demand for personalized healthcare, particularly among individuals requiring continuous health monitoring, has driven significant advancements in sensor technology. Wearable, non-continuous monitoring, and non-contact sensors are leading this innovation, providing novel methods for monitoring vital signs and physiological data in both clinical and home settings. However, there is a lack of comprehensive comparative studies assessing the overall functionality of these technologies. This paper aims to address this gap by presenting a detailed comparative analysis of selected wearable, non-continuous monitoring, and non-contact sensors used for health monitoring. To achieve this, we conducted a comprehensive evaluation of various sensors available on the market, utilizing key indicators such as sensor performance, usability, associated platforms functionality, data management, battery efficiency, and cost-effectiveness. Our findings highlight the strengths and limitations of each sensor type, thus offering valuable insights for the selection of the most appropriate technology based on specific healthcare needs. This study has the potential to serve as a valuable resource for researchers, healthcare providers, and policymakers, contributing to a deeper understanding of existing user-centered health monitoring solutions.

## 1. Introduction

### 1.1. Research Context

In recent years, sensor technologies have revolutionized the landscape of healthcare, becoming essential tools for the acquisition and analysis of physiological and behavioral data with remarkable precision [1,2]. These innovations have not only deepened our understanding of individual health patterns but also opened the way for significant progress in personalized healthcare, enabling interventions that are specifically designed to address the unique needs of each individual [3]. This approach has profound implications for the way healthcare is delivered, particularly in the context of remote monitoring. The ability to collect health data in real time has become increasingly valuable as healthcare systems operate to address the growing demand for long-term, home-based care, particularly as chronic diseases become more prevalent and populations age. In this context, remote monitoring technologies allow individuals to receive continuous care without needing to remain in traditional healthcare settings, such as hospitals or nursing homes, thereby enhancing the quality and accessibility of care while simultaneously reducing the overall burden on healthcare infrastructures [4,5].

One of the most transformative aspects of remote monitoring is its capacity to shift healthcare from a reactive to a proactive model [6]. In traditional healthcare settings, medical interventions typically occur after symptoms manifest or a condition has significantly deteriorated. In contrast, remote monitoring allows for the continuous collection of data, providing healthcare professionals with up-to-date information about a person’s health status. This constant flow of data facilitates the early detection of potential health issues, allowing for timely interventions that can prevent complications before they arise. This proactive approach not only improves health outcomes but also empowers individuals to manage their health more effectively and make informed decisions about their well-being. Furthermore, the widespread adoption of remote health monitoring technologies has far-reaching benefits, not only for individuals but also for the healthcare system as a whole. By reducing the need for frequent in-person visits, remote monitoring optimizes the use of healthcare resources, helping to lower overall costs while improving individual satisfaction with care [7].

Leveraging innovations in sensor design and functionality, technologies such as wearable, non-continuous monitoring, and non-contact sensors are at the forefront of this evolution, offering distinct capabilities for tracking critical health metrics and providing a cost-effective and efficient alternative to traditional on-site clinical monitoring [1,2]. The adaptability of these technologies enables healthcare systems to customize care delivery to specific needs, offering a more personalized and adaptable approach than conventional methods. As adoption continues to rise, these technologies have the potential to not only reduce healthcare costs and improve access to care but also to significantly enhance the overall quality of life of individuals worldwide. However, the diversity in sensor types and the specific contexts in which they excel underscore the importance of understanding their unique characteristics.

### 1.2. State of the Art and Current Trends in Sensor-Based Health Monitoring

Wearable sensor-based health monitoring systems represent a significant advancement in continuous health tracking. By incorporating flexible sensors positioned on various body areas, these systems are capable of capturing real-time physiological and motion data while users are engaged in daily activities. They measure a broad range of vital health signals, including heart rate (HR), blood oxygen saturation (SpO2), blood pressure (BP), respiratory rate (RR), body temperature, and electrodermal activity (EDA). Furthermore, motion sensors such as accelerometers, gyroscopes, and magnetometers are extensively employed to capture activity-related signals [8,9]. Heterogeneous wearable devices, which integrate multiple sensors to measure a variety of signals, are generally preferred over single or dual-sensor modules for effective health monitoring. Their multi-sensor design enables a more comprehensive approach to address diverse healthcare challenges, enhancing their versatility and utility in different clinical and everyday scenarios [6].

Wearable devices are available in various forms and can be seamlessly integrated into textiles, clothing, or accessories, or directly attached to the skin. These devices are broadly categorized based on their placement into three main groups: head, limb, and torso [10]. Head-worn devices include items such as glasses, helmets, headbands, and hearing aids [11]. Limb-based devices are worn on arms, legs, or feet and include smartwatches, bracelets, rings, insoles, and socks [12,13,14]. Wearable devices designed for the torso include options such as biometric shirts, belts, and specialized underwear [15,16]. Furthermore, recent advancements have focused on non-invasive or minimally invasive technologies, leading to innovations including on-skin patches and electronic tattoos, which offer improved user comfort and seamless integration with daily clothing and activities [17,18]. Innovations in stretchable materials have resulted in versatile, tissue-compatible sensors with high biocompatibility, ionic conductivity, and resistance to deformation, making them ideal for body-interfacing health monitoring and motion tracking devices [19,20,21,22].

The integration of low-power electronics, compact sensors, and advanced communication technologies has significantly enhanced the accessibility of wearable systems, facilitating their adoption across various healthcare applications. These include chronic disease management, recovery of motor functions, mental health monitoring, and preventive care [6,8]. By capturing continuous data streams, wearable sensors provide relevant insights into long-term health trends such as cardiovascular health, metabolic activity, and stress levels [8]. In addition, the use of digital biomarkers to develop machine learning algorithms for predicting critical health events, such as cardiac events or falls, marks a particularly promising advancement in wearable technology, enabling early interventions and preventive measures [23]. Recent studies have also highlighted the potential of wearable sensors as digital diagnostic tools, with physiological parameters captured by these devices being effectively used to diagnose a variety of conditions, including cardiovascular diseases [24] and neurological disorders [25,26].

Beside wearable sensors, another type of devices used for health monitoring is represented by non-continuous monitoring sensors. Although these devices require user interaction, unlike wearable sensors that provide continuous tracking, they are designed to monitor vital signs over shorter time intervals. Non-continuous monitoring sensors are particularly useful for episodic assessments, such as capturing detailed cardiac metrics through an electrocardiogram (ECG) during specific times of the day. Many companies, including Linktop [27], A&D Medical [28], and others, provide this type of sensor technology, which is primarily deployed in controlled or clinical settings. Although non-continuous monitoring sensors may not offer the convenience of continuous monitoring, they prioritize data accuracy and reliability, which are essential to make informed clinical decisions. These devices are generally used in more specialized contexts, but their practicality and ease of use also make them suitable for independent use by individuals.

Finally, non-contact sensors constitute a growing frontier in healthcare technology, offering innovative non-invasive solutions that minimize physical interaction with individuals while maintaining effective monitoring [1]. Various non-contact vital sign sensors are available on the market [29]. An example is the mat-type air pressure sensor, which can be integrated into surfaces such as mattresses to continuously detect user movements, pressure distribution, or heartbeats while lying down [30]. Similarly, microbend fiber-optic sensors are a versatile alternative, easily embedded into household items such as cushions, chairs, and beds, providing non-intrusive methods for collecting vital signs [31,32]. Another widely used approach involves camera-based systems, which utilize imaging technologies such as RGB, IR, and thermal cameras to measure HR and RR without physical contact [29]. Additionally, radar-based systems have gained popularity for non-contact vital sign monitoring [33]. Impulse radio ultra-wideband (IR-UWB) radars have been used for complex scenarios such as through-wall HR and RR estimation [34], body movement detection [35], and monitoring multiple targets [36]. A key advantage of IR-UWB radars is their ability to penetrate clothing or blankets and operate effectively in dark environments or at night, as their signals are unaffected by ambient lighting or skin color [37,38,39].

Non-contact technologies are particularly valuable in environments that require strict hygiene or no disturbance to individuals, such as neonatal intensive care units [38], in-home monitoring for elderly individuals [40], and infectious disease facilities [41]. These features make non-contact sensors indispensable in scenarios prioritizing comfort and safety.

### 1.3. Motivation and Problem Statement

Despite the rapid adoption and ongoing advancements in health monitoring sensor technologies, significant gaps remain in evaluating and selecting appropriate sensors for specific healthcare needs [6]. Existing studies aiming to compare these technologies often focus on individual sensor types or specific use cases [42,43,44]. While other research provides broader overviews on various monitoring sensors, such comparative analysis is often insufficiently detailed, leaving healthcare providers and researchers with fragmented insights [8,45,46]. This lack of a comprehensive comparative framework complicates the process of determining which sensors are best suited for specific monitoring tasks, particularly when considering factors such as cost, usability, and integration with other healthcare systems.

Furthermore, the diverse functionalities and inherent limitations of these devices add complexity to the decision-making process. For instance, wearable sensors, while ideal for continuous monitoring, may face issues related to user compliance or data fidelity due to motion artifacts or improper wear [6]. Non-continuous monitoring sensors, although highly accurate, may miss critical real-time health events due to their periodic nature. Non-contact devices, while advantageous for non-invasive monitoring, may struggle with environmental interferences that affect data accuracy [47]. Given the significant potential of these monitoring sensors for human health and the growing interest in this field, addressing the associated challenges and understanding the trade-offs of each sensor type are essential to maximize their effectiveness in healthcare.

Therefore, addressing these issues requires a more holistic evaluation that would better guide stakeholders in identifying technologies that align with specific healthcare needs and support their effective integration into healthcare systems.

### 1.4. Our Contribution

This paper aims to address the aforementioned challenges by conducting a detailed comparative analysis of three types of health-monitoring sensors: wearable, non-continuous monitoring, and non-contact sensors. The objective is to provide healthcare stakeholders with a structured framework to guide the selection of the most suitable monitoring technologies, considering their specific characteristics, performance metrics, and the contexts in which they will be used.

The contributions of this paper include the following:

- An in-depth analysis of selected wearable, non-continuous monitoring, and non-contact technologies, highlighting their strengths, weaknesses, and potential uses in healthcare.
- Practical insights into key factors such as usability, data management, and affordability, to assist healthcare providers in evaluating these devices and making informed choices.
- Suggestions for selecting the most appropriate sensor technology, aimed at enhancing health outcomes through tailored monitoring solutions.

By offering a comprehensive evaluation of these devices, this paper aims to empower stakeholders with the knowledge necessary to effectively integrate user-centered sensor technologies into health practices, driving better care and more efficient resource utilization. The motivations behind the selection of the devices are discussed at the beginning of Section 5.

## 2. Methodology

### 2.1. Criteria for Selecting Sensors

To compare different sensor types for human health monitoring, we conducted an extensive analysis of the sensors available on the market. This subsection outlines the methodology used to identify and select specific sensors based on key criteria, chosen for their relevance and impact on health monitoring applications. Key considerations included the following:

- Sensor Availability: The primary criterion was the availability of sensors on the market and their ease of procurement. By selecting widely available sensors, we ensure the relevance of this analysis to a broad audience and facilitate practical applications in real-world settings.
- Relevance to Health Monitoring: Sensors were selected for their ability to provide accurate and consistent measurements of critical health data, such as vital signs.
- Usability: Usability was considered to assess how easily the sensors could be integrated into daily life, as user comfort and compliance are crucial for successful health monitoring.
- Data Accessibility: Another key factor was the ease of accessing and exporting data from the device. Additionally, the ability to offer real-time or near-real-time tracking is essential for enabling timely interventions and ensuring continuous monitoring.
- Developer Tools for Customization: The availability of Software Development Kits (SDKs) or Application Programming Interfaces (APIs) was a critical factor. These tools allow for integration with custom software, enhancing interoperability with existing platforms and enabling efficient data analysis.
- Cost-Effectiveness: Cost was another crucial criterion, evaluated in relation to the sensor’s functionality. Affordability was essential to promote widespread adoption, especially in resource-constrained settings.
- Findings from Earlier Research: The selection process also considered findings from previous studies assessing the sensor’s performance in health monitoring contexts.

By prioritizing these factors, we aimed to select sensors that meet both technical requirements and the practical needs of individuals and healthcare providers, ensuring a comprehensive approach to human health monitoring.

### 2.2. Categories of Indicators for Sensor Comparative Analysis

All sensors selected from the market were analyzed based on specific indicators. This subsection details the methodology used to systematically evaluate each device. These indicators will guide the comparative analysis presented in Section 4. The following categories of indicators were applied to assess and compare the sensors:

#### 2.2.1. Measured Parameters and Sensor Functionality

This category includes several indicators that assess the overall functionality and operational features of each sensor, with a particular emphasis on the types of parameters measured and the data recording capabilities. The indicators considered in this category are as follows:

- Measured Parameters: An overview of the health data tracked by each sensor.
- Recording Modes: Evaluation of the sensors’ recording modes, including automatic or manual initiation, continuous recording or by specific measurements. This also considers whether users can customize sampling rates, select specific sensor channels, or set a predefined recording duration.
- Memory and Data Storage Capacity: Assessment of the onboard memory capacity, including the sensor’s ability to store data locally for offline use.
- Calibration and Initialization Requirements: Evaluation of the need for initial or periodic calibration to maintain measurement accuracy, as well as the complexity of the calibration process. Consideration is also given to whether the sensors require a warm-up period for accurate functionality.
- Sensor Sensitivity: Evaluation of the factors affecting sensor’s accuracy, such as user movement, body position, fit, vibrations, indirect reflections, and the properties of surrounding materials.
- Environmental Suitability: Review of the sensors’ ability to function in both indoor and outdoor environments, including their resistance to environmental elements.

#### 2.2.2. Sensor Comfort, Design, and Usability

This category evaluates the physical characteristics of the sensors, focusing on comfort, design, and overall usability. These indicators are essential to ensure that sensors are suitable for a diverse range of users and environments, whether worn on the body or integrated into the surroundings. The following indicators are assessed:

- Wearability, Placement, and Comfort: This includes an analysis of where the sensors are worn or placed, their invasiveness, ease of application and removal (particularly for individuals with limited mobility), and comfort during extended use.
- Dimensions, Portability, and Aesthetics: Sensors are evaluated for their size, weight, and portability. An emphasis is also given to their ability to integrate well with everyday clothing or living spaces.
- Physical Constraints: Devices are assessed for their compatibility with different body sizes and the availability of adjustable straps or sizes to ensure a comfortable fit.
- Reusability and Cleaning: This considers the ease of cleaning the sensors and their reusability across different settings and users.

#### 2.2.3. Sensor Platforms and Support Resources

This category evaluates the overall functionality, user-friendliness, and support provided for the sensor’s associated platforms, which can include its proprietary software and mobile applications. Specific indicators in this category are as follows:

- Platform’s Compatibility and Functionality: Evaluation of the proprietary software and mobile app required to operate the sensor, focusing on compatibility with major operating systems, ease of installation, and the features offered. Additional considerations include the ability to manage multiple profiles or devices simultaneously.
- Usability and Accessibility of Platform’s Interfaces: Assessment of the intuitiveness and ease of use of the software and mobile app interfaces, particularly for users with limited technical expertise. Key aspects considered include the clarity of data visualization, ease of settings configuration, simplicity of monitoring, and the availability of language options to ensure broader accessibility.
- Operational Indicators and Alerts: Review of operational status indicators (e.g., LEDs, vibrations, auditory alerts) that support individuals with the use of the device.
- Support and Documentation Quality: Evaluation of the quality of user manuals, online documentation, and technical support provided with the device. This also includes the availability of additional resources for data analysis, as well as the languages in which these materials are offered.
- Availability of Developer Tools: Assessment of the availability of SDKs and APIs that allow developers to integrate the sensor with other systems or customize its features.

#### 2.2.4. Data Transfer, Accessibility, and Export Features

This category evaluates the various methods and features related to the transfer, accessibility, and export of data from the sensor. The following indicators are considered:

- Data Transfer: Analysis of the methods used to transfer data from the sensor to its associated platforms, focusing on the ease and efficiency of this process.
- Data Accessibility: Evaluation of how easily users can access the data, in real time or post-recording, as well as the ability to access the data independently of the device’s operational status.
- Raw Data Export: Assessment of whether raw data can be exported, the ease of the export process, and the supported file formats.
- Reporting Features: Evaluation of the sensor’s platform ability to generate automated reports, including graphs and summaries.

#### 2.2.5. Battery Performance and Power Management

This category evaluates the sensor’s battery efficiency and power management features, highlighting their critical role in ensuring effective long-term monitoring. The following indicators are considered:

- Battery Life: Assessment of the sensor’s battery life, focusing on its duration before a recharge is needed.
- Charging Method: Evaluation of charging methods and how practical and convenient they are for users.
- Recharge Time: Assessment of the time required for the sensor’s battery to fully recharge.
- Power-Saving Features: Analysis of the sensors’ energy-saving modes designed to extend battery life.

#### 2.2.6. Cost Considerations

This category examines the financial aspects associated with each sensor, focusing on the following key factors:

- Initial Price: The purchase price of the sensor, which represents the upfront investment required for its use.
- Subscription Requirements: Analysis of any recurring costs for accessing the sensor’s software, services, or data, which could impact long-term affordability.
- Additional Costs: Assessment of any supplementary costs such as battery or sensor replacements, software updates, or maintenance.

## 3. Overview of Selected Sensors

A targeted selection of five sensors was chosen from the market based on their relevance to health monitoring applications. These devices can be classified into three main categories based on their designs, functionalities, and interaction modes: wearable sensors, non-continuous monitoring sensors, and non-contact sensors. The selection process emphasized a diversity of options to ensure a thorough comparative analysis.

### 3.1. Wearable Sensors Selected for This Study

In the category of wearable sensors, we included devices with specific characteristics that distinguish them from non-continuous monitoring and non-contact sensors. Wearable devices are designed to integrate into daily life through clothing, accessories, or direct skin attachment, and are often intended to be worn continuously. Therefore, these sensors can provide continuous, uninterrupted monitoring of vital and motor parameters, aiding in the detection of subtle physiological changes that non-continuous devices might miss. Additionally, wearable devices can also monitor users during dynamic movements, a condition that poses challenges for non-contact devices, which typically require users to remain stationary for accurate readings.

Given these characteristics, examples of wearable sensors examined in this study include EmotiBit, OpenGo Sensor Insoles, and NexRing.

#### 3.1.1. EmotiBit

**Figure 1:**
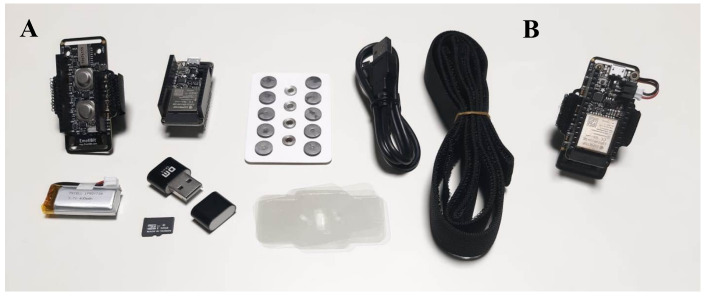
EmotiBit components and assembly. (**A**) The complete EmotiBit bundle, including the EmotiBit MD board, Adafruit Huzzah32 Feather, 400 mAh lithium ion battery, high-speed microSD card, microSD card reader, electrode kit, Emoti-genic covers, micro USB cable, and Emoti-stretch straps of varying lengths. (**B**) The fully assembled EmotiBit.

EmotiBit (EmotiBit, Reno, Nevada, United States) qualifies as a wearable sensor due to its ability to be worn on various body locations during daily life, while providing continuous monitoring of biometric signals (Figure 1). Its comprehensive sensor set, including a three-wavelength photoplethysmography (PPG: red, infrared, and green), an EDA sensor, a temperature sensor, and a nine-axis inertial measurement unit (IMU: six-axis accelerometer, gyroscope, and three-axis magnetometer), enables the detection of high-quality emotional, physiological, and movement data, even during dynamic activities. EmotiBit is open-source and Arduino compatible, and offers wireless data streaming to any platform, empowering users to explore their own health and wellness [48].

A pioneering validation study conducted by Montgomery and colleagues compared EmotiBit with a gold-standard physiological sensing device, the Brain Products system, demonstrating that EmotiBit provided comparable signals for HR, EDA, and accelerometer data [49]. In the context of emotional and physiological research, EmotiBit has been employed in various studies. Loboscopo used EmotiBit to assess the effects of dancing with a partner on emotional and biophysical states [50], while Morris and collaborators employed it to collect PPG and EDA data to better investigate the cognitive load and explicit awareness of wearable devices, critical factors for the long-term adoption of such technologies [51]. EmotiBit was also used to detect physiological data in specific populations, such as autistic employees working in a virtual environment [52] and adults with mild cognitive impairment engaged in activity programs complemented by virtual reality [53]. Other studies have used the motion data derived from EmotiBit to explore an alternative method for screening spine diseases, such as scoliosis and sagittal imbalance [54], or to obtain real-time movement analysis during physical activity interventions supported by augmented reality for bedridden patients [55]. Beyond human use, EmotiBit has also been applied to monitor physiological data from animals, as demonstrated by Olivaz et al. [56]. These diverse applications demonstrate the versatility and potential of EmotiBit in various research fields, further underscoring its suitability as a wearable sensor.

#### 3.1.2. OpenGo Sensor Insoles

**Figure 2:**
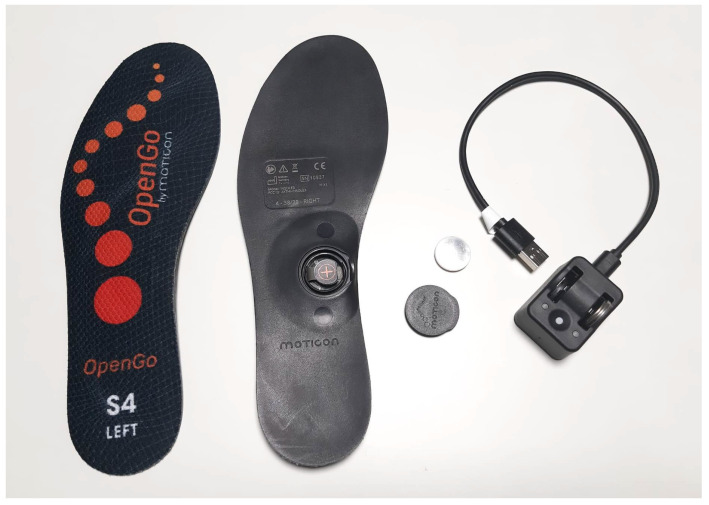
OpenGo Sensor Insoles. The figure presents a pair of OpenGo Sensor Insoles, showing both top and bottom views, with the battery lid and coin cell battery removed, along with the charging dock featuring an inserted battery for recharging.

OpenGo Sensor Insoles (Moticon, Munich, Germany) represent an advanced, fully integrated wearable device for capturing and analyzing human foot dynamics (Figure 2). Equipped with 16 capacitive pressure sensors, a three-axis accelerometer, and a three-axis gyroscope, the insoles deliver high-resolution data on plantar pressure distributions, foot acceleration, and rotational dynamics in three axes [57]. Their design ensures flexibility and functionality, as they are intended for use inside a shoe, allowing for accurate data collection during natural and dynamic movements without disrupting normal activity. Their standalone system eliminates the need for external devices or cables, further enhancing usability and supporting uninterrupted monitoring. These features make OpenGo Sensor Insoles well suited for applications in movement research, sports science, and other dynamic settings.

OpenGo Sensor Insoles have proven to be a reliable tool for gait analysis, offering accuracy comparable to a force plate system. A 2015 study [58] confirmed their validity in healthy individuals, supporting their use in research trials requiring detailed step-by-step and long-term gait analysis. The insoles have also been effective in detecting pathological gait patterns [59] and in distinguishing between groups with different gait characteristics [60,61].

The versatility of OpenGo Sensor Insoles extends to various applications. They have been used to measure ground-to-foot forces during basic activities before and after total knee arthroplasty [62], monitor lower-limb loads during daily activities [63], and estimate continuous body weight changes through machine learning algorithms, offering potential in health conditions involving weight fluctuations such as edema [64]. In occupational health, the insoles have been used to assess fall risks in construction workers [65], and their ability to track gait responses in older adults under challenging environmental conditions highlights their suitability for real-life scenarios [66].

#### 3.1.3. NexRing

**Figure 3:**
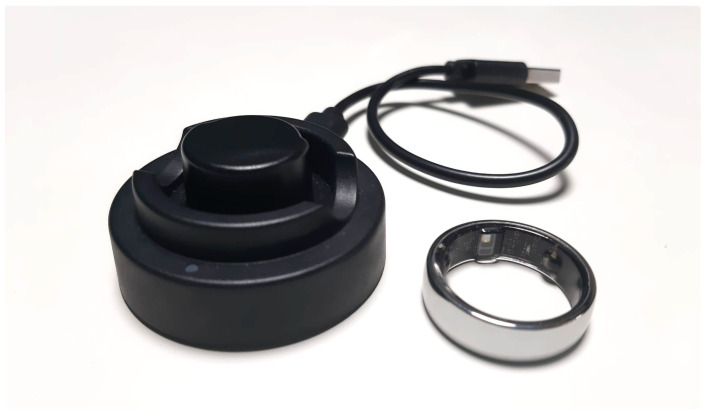
NexRing. The figure illustrates NexRing with its wireless charging dock.

NexRing (Linktop Technology Co., Ltd., Xiamen City, China) can be classified as a wearable sensor due to its advanced design as a digital smart ring (Figure 3). Its compact, unobtrusive form and 24/7 wearability ensure that it can be worn comfortably throughout the day without interfering with daily activities, allowing for the continuous monitoring of vital metrics and activity levels. While no specific scientific literature currently exists for this device, its practical design and functionality clearly demonstrate its suitability for long-term health monitoring, aligning it with the core characteristics of wearable sensors [67].

Unlike wrist-based alternatives, NexRing offers superior comfort and enhanced data accuracy by utilizing the finger’s pulse signal, which is stronger and more reliable than wrist-based measurements [68,69,70]. The functionalities of NexRing are similar to those of other health-tracking rings, such as the Oura Ring, which have been shown to be effective in monitoring various health parameters [71,72]. However, unlike the Oura Ring, which requires a recurring monthly payment for access to its app, NexRing provides an affordable solution by offering full access to its features without ongoing fees, presenting a cost-effective option for prolonged use.

### 3.2. Non-Continuous Monitoring Sensor Selected for This Study

Non-continuous monitoring sensors can be distinguished by their ability to provide only intermittent data through single-point snapshots, offering instantaneous or interval-based measurements, often requiring user interaction. These devices are particularly useful for periodic data collection and can be sufficient for point-of-care diagnostics or episodic monitoring. However, they lack the capability to monitor ongoing changes dynamically.

**Figure 4:**
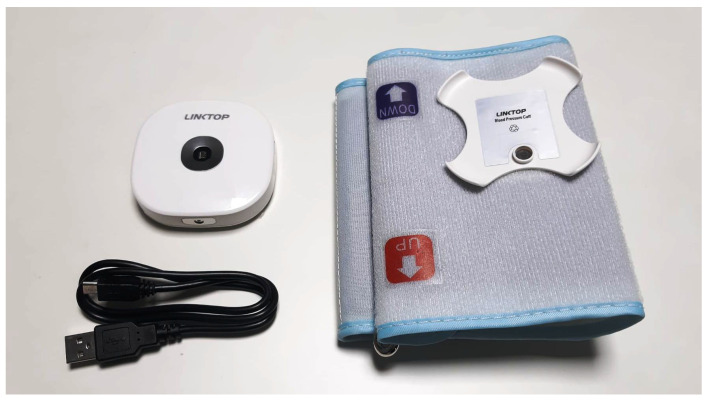
The 6-in-1 Remote Health Monitor. The figure presents the 6-in-1 Remote Health Monitor with its cuff for blood pressure measurements and a charging cable.

An example of a non-continuous monitoring sensor included in this study is the 6-in-1 Remote Health Monitor (model HC-03, Linktop Technology Co., Ltd., Xiamen City, China), a portable health tracking device designed to measure various critical health parameters (Figure 4). The device is CE-certified as a Class 2A medical device, ensuring that its measurement data comply with medical-grade accuracy standards [73]. It is equipped with advanced sensors, providing precise measurements of systolic and diastolic BP, SpO2, and body temperature. In addition, the ECG function offers valuable cardiac data.

Despite its comprehensive set of measurements, the 6-in-1 Remote Health Monitor lacks the ability to continuously monitor dynamic, real-time changes in these health parameters. Its design is focused on providing snapshot data rather than offering ongoing monitoring, and measurements require user interaction to initiate the data collection. This positions it within our category of non-continuous monitoring sensors.

The device is primarily intended for use by medical professionals, even if it is also suitable for use by independent individuals, offering an intuitive interface that makes it accessible for non-professional users. Although no literature is provided, the device has been selected due to its all-in-one design with accurate and reliable measurements.

### 3.3. Non-Contact Sensor Selected for This Study

Devices classified under the category of non-contact monitoring are those designed to provide vital sign measurements and detect movements without requiring physical contact. This category includes advanced technologies such as IR-UWB radars, which can monitor health parameters and movements without direct physical interaction with the user. These radar systems are capable of functioning effectively even through walls, clothing, or blankets, as well as in low-light or nighttime conditions.

**Figure 5:**
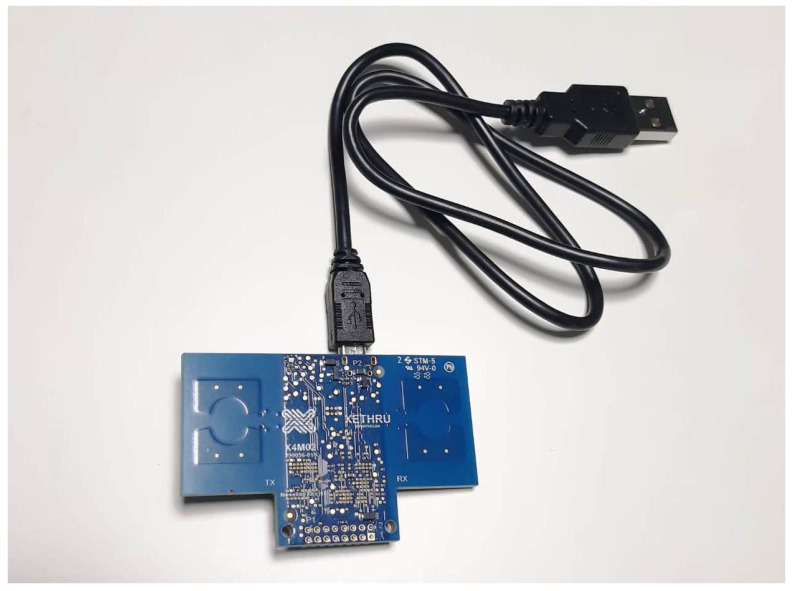
XeThru X4M200 respiration sensor. The figure illustrates the XeThru X4M200 respiration sensor, along with a micro USB cable for powering the device.

An example of a non-contact sensor analyzed in this study is the XeThru X4M200 respiration sensor, an industrial-grade, non-contact radar device from NOVELDA (Oslo, Norway) (Figure 5).

Powered by the XeThru X4 system-on-chip, the XeThru X4M200 respiration sensor is specifically designed for the precise monitoring of respiration and movement without physical contact [74]. The XeThru X4 is a complete IR-UWB radar system that uses pulse-Doppler signal processing to transmit and receive coherent pulses via built-in antennas. The system emits pulses at a configurable pulse repetition frequency of 15.875 MHz, with a 27 MHz oscillator controlling the transmission. The time between pulses determines the radar’s range, and reflected signals are analyzed to detect movement and estimate velocity using the Doppler Effect. Operating in the frequency range of 6.0–10.2 GHz (6.0–8.5 GHz for low-frequency bands, 7.25–10.2 GHz for high-frequency bands), the X4M200 sensor provides highly accurate respiratory measurements up to 5 m, even through solid barriers. Its programmable detection range and four selectable respiration profiles, tailored to different subjects (adults, children, and infants), make it suitable for various applications during both daytime and nighttime monitoring. Compact, cost-effective, and easy to install, the X4M200 sensor combines advanced technology with practical versatility for applications requiring precise contactless sensing.

The XeThru X4M200 respiration sensor plays a key role in various research projects related to non-contact vital sign monitoring. Saeed and colleagues used the sensor to track breathing patterns in real time, with future applications intended for patients with respiratory illnesses [75]. Qiao demonstrated its accuracy in measuring cardiac motion in home healthcare [76], while Xu developed a signal processing method to improve the detection accuracy in complex conditions [77]. Hossain addressed the challenge of obtaining accurate breathing signals from non-stationary subjects, such as during sleep [78], and Sjöman utilized the radar in an interactive environment where the breathing pattern of the individual was synchronized with a dynamically modifiable space, promoting relaxation and mindfulness [79]. Additionally, the radar has been applied in daily activity recognition and fall detection, offering promising results for senior assistance and health monitoring in smart homes [80,81,82]. In outdoor environments, Jing explored its use while mounted on a drone for detecting respiration in injured subjects [83].

## 4. Comparative Analysis of Selected Wearable, Non-Continuous Monitoring, and Non-Contact Sensors

The five sensors selected for this study were compared based on the key indicators discussed above. This comparative analysis led to important findings, which are presented in this section, along with summary tables highlighting the results.

### 4.1. Comparison of Measured Parameters and Sensor Functionality

The comparison of sensors across their ability to measure key health parameters reveals significant differences (Table 1). EmotiBit stands out for its comprehensive measurement capabilities, tracking a wide array of physiological metrics. The PPG sensor allows for the extraction of several vital parameters, including interbeat interval (IBI), HR, heart rate variability (HRV), SpO2, RR, and fluctuations in BP. These metrics provide valuable insights into autonomic function, cardiovascular health, and respiratory patterns [84,85]. The EDA sensor can measure galvanic skin responses, reflecting sympathetic nervous system activity associated with emotional and cognitive arousal [70,86]. Furthermore, EmotiBit features a medical-grade thermopile with a resolution of 0.001 °C, ensuring precise measurements of perspiration, body temperature, and overall physiological activation, thereby offering further insights into the body’s responses to environmental and emotional stimuli. The device also includes a nine-axis IMU that allows the detection of a wide range of movement-related metrics, offering detailed information on activity patterns [87]. Moreover, the combination of accelerometer and PPG signals can be used to estimate sleep stages [88,89]. A recent study demonstrated EmotiBit’s strong reliability across multiple sensing domains, showing high correlations with gold-standard devices for key measurements, including HR, EDA, and accelerometer data [49].

In contrast, OpenGo Sensor Insoles are specifically designed to measure parameters such as pressure, acceleration, angular rate, total force, and center of pressure (COP), which are crucial for evaluating gait and balance performances [59,61,65,66]. Each insole is equipped with 16 capacitive pressure sensors (range: 0–50 N/cm²; resolution: 0.25 N/cm²), a three-axis accelerometer (range: ±16 g; resolution: 0.488 mg/LSB, 16-bit), and a three-axis gyroscope (range: ±2000 dps; resolution: 70 mdps/LSB, 16-bit). A total force validation study conducted by Moticon, comparing Moticon Sensor Insoles to force plates during normal walking, revealed that the highest accuracy was achieved with calibrated insoles, with an average normalized mean absolute error of 2.04% and an average peak error of 6.90% [90]. Although OpenGo Sensor Insoles have shown some discrepancies compared to force plates in dynamic activities such as running and jumping, they exhibited strong agreement with force plate data for cycle characteristics and temporal parameters during gait and jumping tasks [91]. In balance tasks, discrepancies were noted in medio-lateral stability measurements [91]; however, accuracy improved with higher vertical forces and the activation of additional pressure cells [92].

NexRing is a comprehensive health monitoring device that monitors a range of vital signs, including HR (with updates every five minutes), HRV, HR dip, RR, SpO2, and finger temperature, along with activity-related metrics such as steps, distance, and calories burned. It also offers detailed sleep tracking, including sleep stages, and personalized scores for readiness and sleep quality, although vital sign measurements, except for HR, are only available during sleep.

The 6-in-1 Remote Health Monitor measures systolic and diastolic BP using oscillometric technology. Systolic BP is measured within a range of 60–230 mmHg, and diastolic BP within a range of 40–130 mmHg, both with a tolerance of ±3 mmHg. The device employs pulse oximetry to provide SpO2 and HR measurements. HR measurements are within the range of 40–180 bpm, with a tolerance of ±5%; while SpO2 is measured between 70 and 100%, with a tolerance of ±4% for 70% to 80% SpO2, ±2% for 80% to 90% SpO2, and ±1% for 90% to 100% SpO2. Body temperature is assessed using infrared technology and ranges from 28 to 42 °C, with a tolerance of ±0.2 °C for temperatures between 35–42 °C and ±0.4 °C outside this range. The 30-second ECG recording provides additional parameters such as average HR, HRV, RR, and maximum and minimum IBI. The ECG function utilizes single-lead measurements with a sensitivity error ≤±5% and a sampling rate of 512 Hz, ensuring accurate and reliable cardiac data.

Finally, the XeThru X4M200 respiration sensor focuses on measuring respiratory and movement data, including RR, breathing patterns, distance to the target, and movement history. Breathing data are detected only when the individual is stationary, typically while lying down or sitting. Additionally, the X4 module can be used to derive health metrics such as HR, HRV, and sleep stages [93]. A recent study comparing the XeThru X4M200 respiration sensor with reference devices reported an average mean absolute error of 0.65 for respiration detection and 1.32 for HR detection, using a respiration belt and an ECG sensor as benchmarks [77].

The devices also exhibit differences in terms of recording modes and functionality (Table 2). All devices, with the exception of the 6-in-1 Remote Health Monitor, are designed for continuous monitoring, while the 6-in-1 Remote Health Monitor supports non-continuous monitoring, allowing users to track specific health metrics through specific measurements. Additionally, all devices, except for NexRing, require manual initiation for data recording. In contrast, NexRing automatically records data when worn and synced with its companion app. The only device that offers the option to select the sampling rate is the OpenGo Sensor Insoles, while EmotiBit allows for an increased PPG rate (100 Hz) through a specific firmware variant, making it suitable for applications requiring higher-resolution data, such as HRV analysis.

When using OpenGo Sensor Insoles, users can configure the sample rate and select channels for data recording. Channel selection can be made using predefined setups or by manually choosing specific channels. Additionally, the insoles offer three operational modes tailored to different use cases: (i) Live Capture Mode, which enables direct data transmission from the insoles to the OpenGo software using the mobile app as a hub, ideal for indoor lab environments as it does not store data on the onboard memory; (ii) Record Mode, which stores data directly on the insoles’ onboard memory, suitable for field use; and (iii) Smart Recording Mode, which activates recording only during user activity making it ideal for long-term monitoring. Finally, a timer function for recording is available only for OpenGo Sensor Insoles. NexRing also offers predefined recording times, but only during app-guided workouts and mindfulness sessions. A predefined recording duration could be useful when monitoring specific events, such as the effect of a medication or sessions of physical activity, eliminating the need for the user to manually stop the recording. Sessions with a predefined duration would also optimize battery life and memory management.

The XeThru X4M200 allows data recording based on four distinct respiratory profiles, tailored for specific use cases. Each profile corresponds to a defined RR range (e.g., 8–30 breaths per minute for adults, 15–65 breaths per minute for infants and children) and a duration of the Slow and Fast Pulse-Doppler algorithms (6 and 20 s, respectively, for adults; 6 and 15 s, respectively, for infants and children). The profiles also feature a specific detection zone, configurable from a minimum of 0.40 m to a maximum of 5 m, with an adjustable resolution of 1 cm and a step size of 5.14 cm. The device also allows users to adjust sensitivity, and it features noise map control with three modes: default noise map, stored noise map, and an adaptive noise map that dynamically adjusts to environmental changes. Furthermore, users can configure the radar’s frequency band (low: 6.0–8.5 GHz, or high: 7.25–10.2 GHz). Finally, the X4M200 radar can detect presence and vital signs through lightweight materials, such as blankets or clothing, adding versatility for applications such as sleep monitoring.

In terms of memory capacity, the devices with the largest storage appear to be EmotiBit and NexRing. Regarding calibration and initialization requirements, it should be noted that Montgomery [49] recommends a 5 min period before starting data collection with EmotiBit, allowing the sensors to settle for accurate readings. Before recording with OpenGo Sensor Insoles, it is required to warm up the insoles for few minutes and take some steps to facilitate the automated zeroing process, which eliminates pressure signal drifts caused by temperature variations or wear. Additionally, an initial user-specific weight calibration is required to enhance the accuracy of the total force readings of the insoles. This process involves a 1.5 min routine of specific movements (e.g., walking, standing still, and shifting body weight), slowly performed and step-by-step guided.

The accuracy of each sensor can be influenced by specific factors. For example, proper attachment of the EmotiBit to the body is critical to minimize motion artifacts, particularly since the PPG signal is highly susceptible to disturbances. However, the device should not be worn too tightly, as this could constrict underlying vascular systems, potentially affecting blood flow and distorting the signal [84,94]. Additionally, the sensor’s accuracy may be influenced by the wearer’s body position [78,84]. Regarding OpenGo Sensor Insoles and NexRing, data accuracy may be impacted by improper sizing (e.g., incorrect fit), and, for the 6-in-1 Remote Health Monitor, measurement accuracy may be affected by an incorrect device placement, subject’s conditions (e.g., sweat or creams on the forehead can affect body temperature readings, while wet fingers can influence ECG accuracy), movement, or improper posture (e.g., not aligning the BP cuff with the heart level). The performance of the XeThru X4M200 can be influenced by vibrations, indirect reflections, surrounding surface material properties, and nearby metallic objects.

Finally, most devices appear suitable for both indoor and outdoor use. The 6-in-1 Remote Health Monitor is specifically designed for indoor environments, particularly in healthcare settings or homes, where measurements should be taken in stable conditions. For added durability, users can attach a protective case to the EmotiBit for safer use.

### 4.2. Assessment of Sensor Comfort, Design, and Usability

Selected sensors feature significant differences in terms of comfort, design, and usability, as described in Table 3. EmotiBit offers great flexibility in placement and orientation due to its adjustable straps, allowing it to be used on various body parts, from a child’s wrist to an adult’s head. Placement examples for EmotiBit include the middle finger [49], the ankle [55], and the upper arm [51]. On the other hand, OpenGo Sensor Insoles and NexRing are intended for use on specific body parts. For optimal use, NexRing is recommended to be worn on the index finger of the non-dominant hand, with the sensors facing the palm, while avoiding pairing it with other rings to prevent discomfort or damage. NexRing is durable, waterproof, and suitable for daily activities such as showering or hand washing, although it is best avoided during activities involving friction or when handling heavy metal, ceramic, or stone objects to prevent scratches. OpenGo Sensor Insoles are easy to put on and provide a natural fit with minimal interference with natural movement. Some users may experience mild initial discomfort when wearing the insoles, particularly in the elevated medial midfoot area of the insoles, which houses the electronics and power supply. Both insoles and the ring seamlessly integrate into daily clothing, while EmotiBit, although its stretchable design makes it easy to attach, is less discreet and may not blend smoothly with everyday attire, appearing more as an additional accessory. However, to enhance the integration and reduce the visibility of EmotiBit, users can customize a strap-and-go case to hold the device.

The 6-in-1 Remote Health Monitor can measure multiple vital parameters from specific body locations with minimal invasiveness. Body temperature is taken from the center of the forehead, maintaining a distance of 1–2 cm, while BP is measured with the cuff positioned 1–2 cm above the left elbow joint. To facilitate proper use, the BP cuff is equipped with clear indicators for correct placement. SpO2 and ECG readings are obtained by interacting directly with the device. For SpO2, the left middle finger should rest on the sensor with the fingertip touching the probe, while, for ECG measurements, the device is held in the left hand, with the thumb placed on the oxygen sensor and the other fingers touching the metallic label on the back. The right hand is then placed on the body temperature sensor, with the two hands not touching each other [95]. While practical for most measurements, the ECG function requires the use of both hands, which may pose difficulties for individuals with limited hand mobility or those without assistance.

The XeThru X4M200 respiration sensor is entirely non-contact, and can be positioned on surfaces including desks, walls, or ceilings, and even placed behind obstacles such as walls or ceilings, depending on the material properties. A potential constraint to consider when positioning the device is the stability of the surface on which it is placed. Additionally, its small size and unobtrusive design make it ideal for integration into home environments.

All selected devices are compact and portable, and, except for the radar, offer adaptable solutions for users with different body sizes. OpenGo Sensor Insoles, although available in a wide range of sizes (EU 32/33 to 48/49; US 1/2 to 12½/13½) and covering 98% of adult sizes [96], are not suitable for users with custom orthotics or with a body weight exceeding 120 kg. Additionally, the product guidelines caution against use with open wounds, unhealthy foot skin, or severe gait impairments. The insoles must be worn with socks to avoid direct skin contact and are not suitable for wet conditions (e.g., rain or excessive foot sweat) or activities that may excessively bend or twist the insole, such as driving or climbing ladders. Regarding reusability and cleaning, all devices are designed for reuse by different users or in various settings, although cleaning methods vary.

### 4.3. Analysis of Sensor Platforms and Support Resources

Table 4 compares selected devices across various categories related to sensor platforms and support resources. Devices such as EmotiBit and XeThru X4M200 respiration sensor offer software-based solutions: the EmotiBit Oscilloscope and DataParser for EmotiBit, and the XeThru Explorer for the XeThru X4M200. The EmotiBit Oscilloscope is designed for the real-time visualization of signals, including PPG, HR, EDA, accelerometer, gyroscope, and magnetometer data, as well as temperature and frequency of skin conductance responses. It also allows users to record data onto the onboard SD card, manage power modes to conserve the battery, annotate data for further analysis, and stream data. The DataParser complements this by processing recorded data offline, allowing raw files to be converted into parsed data files for detailed analysis. XeThru Explorer enables the visualization and management of sensor data, offering advanced real-time metrics such as RR, breathing patterns, movement history, and radar data (e.g., pulse-Doppler data and baseband signals). It also allows users to select suitable respiration profiles, adjust settings, and record or replay measurements.

In contrast, NexRing and the 6-in-1 Remote Health Monitor operate exclusively via dedicated mobile apps. The NexRing App allows users to track daily activity, sleep patterns, and health metrics over time, presenting data through graphs and enabling customization with tags. The Linktop Health Monitor App for the 6-in-1 device records health data and provides real-time visualization of measurements such as SpO2, HR, and ECG waveforms, along with parameters of HRV and RRI. It also features historical data visualization and data export capabilities.

OpenGo Sensor Insoles provide the most comprehensive platform, offering both desktop software (OpenGo Software) and a mobile app (OpenGo Mobile App) [97]. The mobile app manages insole configuration, initiates and stops measurements, previews live data, and calibrates force readings, while the desktop software provides data analysis, customizable dashboards, and data export options. Furthermore, for OpenGo Sensor Insoles, as well as for NexRing, the 6-in-1 Remote Health Monitor, and XeThru X4M200, only one device can be managed at a time. With EmotiBit, instead, it is possible to connect multiple devices individually by using separate Oscilloscope instances.

Regarding usability and accessibility, the platforms of OpenGo Sensor Insoles, NexRing, and the 6-in-1 Remote Health Monitor feature intuitive and user-friendly interfaces. OpenGo Software supports customizable dashboards, while the Linktop Health Monitor App provides clear measurement blocks, with seconds left until completion of ECG recordings and physiological reference values for some health parameters. NexRing offers the effortless tracking of health trends via detailed graphs. In contrast, EmotiBit and XeThru X4M200 may require technical expertise for data interpretation as both devices focus on signal trends rather than easily interpretable parameters, which may be challenging for non-expert users. Additionally, while platforms’ installation procedures are relatively straightforward and includes step-by-step guidance for most devices, installing EmotiBit’s software and firmware, as well as assembling the device and waking it from the Sleep Mode—despite being thoroughly detailed in the provided documentation [98]—may present challenges for users with limited technological proficiency or reduced manual dexterity.

All devices are equipped with operational indicators and alerts to assist users. OpenGo Sensor Insoles excel in this area, offering various features such as readiness indicators for zeroing (displaying different colors to indicate whether the process is successful), pre-measurement pop-ups for assigning measurement labels, and biofeedback alerts via beeps or flashes to facilitate synchronization with other systems. However, it is worth noting that associated platforms do not provide users with comprehensive guidance regarding proper positioning during measurements. Such guidance could be particularly beneficial for complex procedures, such as for the ECG reading with the 6-in-1 Remote Health Monitor, that requires precise hand placement on the device. In this case, the only on-screen instruction advises users to ensure contact with the lead films on the back of the device, while also displaying the signal quality. The user manual for the 6-in-1 Remote Health Monitor suggests that an inverted waveform during ECG readings may indicate improper hand positioning, but these observations rely on the user’s ability to recognize errors and make necessary adjustments, which may demand professional knowledge. Similarly, the device notifies whether a BP measurement is initiated without the device being inserted into the cuff, but it does not confirm whether the cuff is correctly positioned.

The devices are accompanied by documentation and user instructions; however, only EmotiBit and OpenGo Sensor Insoles provide dedicated libraries for advanced data processing. Language support also differs; the resources of EmotiBit and XeThru X4M200 are available exclusively in English, while NexRing and the 6-in-1 app support multiple languages. OpenGo offers multiple languages only for its software, as the mobile app and documentation are available exclusively in English. Finally, the devices provide developer tools to support custom solutions. While EmotiBit offers open-source software, the other devices provide SDKs or APIs for integration and advanced customization.

### 4.4. Evaluation of Data Transfer, Accessibility, and Export Features

All the devices support automatic data transfer to their respective platforms, though with different methods (Table 5). EmotiBit uses Wi-Fi, NexRing and the 6-in-1 Remote Health Monitor rely on Bluetooth, while the XeThru X4M200 requires a micro USB connection. EmotiBit offers additional flexibility by supporting OSC, UDP, and LSL protocols for transmitting data from the software to user-defined output channels.

Data transfer from OpenGo Sensor Insoles to the mobile app is via Bluetooth, while that from the mobile app to the desktop software is supported by Wi-Fi, with the transfer time ranging from seconds to minutes depending on the amount of data and recording mode. This transfer can occur automatically or by manual initiation, depending on the user settings for recording data.

Data accessibility is robust across all devices, with data recordings remaining accessible after disconnection. However, for OpenGo Sensor Insoles, this applies only if data has been transferred to the software and, for NexRing, data can only be viewed through the mobile app.

Regarding raw data export, significant differences exist among the devices. EmotiBit and OpenGo Sensor Insoles allow raw data export in .csv and .txt formats, respectively. EmotiBit’s data is fully user-owned and stored on the onboard SD card. The simplest method to access the data is by connecting the SD card to a computer. Raw data are saved as a single file per session, along with an information file (.json) detailing recording settings. Parsed data files (.csv) specific to individual sensors of EmotiBit can also be generated using the DataParser tool. OpenGo Sensor Insoles’ raw data can be exported via their software, and, in total, each raw data file provides one timestamp channel and the following sensor channels for each insole: 16 pressure channels, 3 acceleration channels, 3 angular rate channels, 1 total force channel (computed onboard), and 2 COP channels in the X and Y directions (computed onboard). Also, the X4M200 sensor allows for raw data export. Such data, in .dat format, are automatically stored in a user-defined directory after recording. The desktop software allows users to configure file storage preferences and data segmentation by time or file size. By default, the XeThru module generates 17 baseband data frames per second, offering raw I/Q (In-Phase and Quadrature) or Amplitude/Phase data for customized analyses. Additional data types include Pulse Doppler (Float and Byte), Noise Map (Float and Byte), and Generic Data (Float, Byte, and String), along with application-specific outputs such as respiration, sleep, and presence data, all in .csv format. In contrast, NexRing and the 6-in-1 Remote Health Monitor do not support raw data export.

Reporting capabilities also vary significantly. Only OpenGo Sensor Insoles and the 6-in-1 Remote Health Monitor generate automated reports. OpenGo offers extensive reporting options, including .pdf and .xlsx reports tailored to different analyses, such as gait and balance assessments. These reports include both graphical and numerical representations and allow for the comparison of multiple measurements [99]. The 6-in-1 Remote Health Monitor generates basic reports for individual health parameters. Reports for metrics such as BP, HR, and SpO2 include only numerical values and encompass all recorded sessions. The ECG report, instead, combines numerical data with waveform graphs, but only supports one measurement per report.

### 4.5. Examination of Battery Performance and Power Management

Table 6 provides an overview of how battery-related features vary across the selected devices. Wearable devices demonstrate significant differences in battery life, with NexRing emerging as the most durable, followed by OpenGo Sensor Insoles and EmotiBit. The battery life of OpenGo Sensor Insoles is influenced by factors such as operation mode, sampling rate, and the number of active sensor channels. For data stored on the onboard memory of OpenGo Sensor Insoles, battery life is generally not the limiting factor; instead, the available memory determines the recording duration. The 6-in-1 Remote Health Monitor features a 400 mAh rechargeable lithium battery designed for up to 500 charge cycles under optimal conditions, while the XeThru X4M200 respiration sensor does not feature an internal battery and relies entirely on external power sources. It can be powered either through a micro USB cable or via pins 1 and 2 on the 16-pin interface connector. If both power sources are connected simultaneously, the USB connection takes precedence.

In terms of charging methods, EmotiBit and the 6-in-1 Remote Health Monitor rely on a simple micro USB connection to a computer or a compatible power source, offering a practical and portable solution for daily recharging. NexRing also requires a USB charging connected to a wireless charging dock where the ring is placed. For NexRing, it is recommended to maintain the battery level above 30% before bedtime for uninterrupted sleep tracking. Similarly, OpenGo Sensor Insoles use a charging dock; however, recharging requires removing the coin cell batteries from the insoles and placing them in the dedicated charging slot, which connects via USB to the power source.

Recharge time also highlights key differences between the devices. NexRing is the fastest to recharge, followed by EmotiBit and OpenGo Sensor Insoles, with the 6-in-1 Remote Health Monitor taking the longest. Most devices incorporate power-saving features to enhance energy efficiency. EmotiBit offers multiple options, including a Low Power Mode, for data recording without real-time transmission; a Wi-Fi Off Mode, which disables the onboard Wi-Fi shield for extended recording sessions; a Sleep Mode, where the device stops all tasks and enters a low-power state ideal for short inactivity; and a Hibernate switch for maximum power conservation during prolonged inactivity. For OpenGo Sensor Insoles, Sleep Mode is recommended during extended periods of inactivity, and the optimizing sensor setup further conserves battery life and maximizes storage efficiency. NexRing also features a Power Saving Mode to preserve battery capacity and prevent deterioration during non-use. Similarly, the 6-in-1 Remote Health Monitor includes a low-performance mode designed specifically for ECG measurements, optimizing power consumption during operation. These variations in battery life, charging methods, and power management reflect the distinct design priorities and intended use cases for each device.

### 4.6. Comparison of Costs and Economic Considerations

Table 7 presents a comparison of the financial aspects associated with each sensor, focusing on initial price, subscription requirements, and additional costs. All sensors do not require subscriptions, making them cost-effective in terms of ongoing expenses. OpenGo Sensor Insoles have the highest initial cost and may necessitate significant additional expenses for SDKs. NexRing, the 6-in-1 Remote Health Monitor, and XeThru X4M200 sensor offer moderate initial prices with no clear additional costs. Firmware updates for NexRing are provided for free through the mobile app, while firmware upgrades for XeThru X4M200 are available through new versions of the software. EmotiBit offers a similarly affordable and cost-effective option, available as a complete bundle.

## 5. Discussion

### 5.1. Interpretation of Findings

This paper aimed to conduct a comprehensive comparative analysis of five health monitoring sensors available on the market, with the objective of providing healthcare stakeholders with a structured framework for selecting the most suitable technologies for specific health monitoring needs. The sensors were chosen for their potential to effectively monitor the health status of individuals, particularly those who are vulnerable or require tailored, personalized care solutions.

EmotiBit was selected for its ability to capture high-quality emotional, physiological, and movement data while ensuring that data ownership remains with the user. This feature makes it particularly well suited to personalized health monitoring scenarios. The inclusion of OpenGo Sensor Insoles addressed the critical need to monitor mobility-related health risks, such as falls and gait disorders, providing precise insights into balance and locomotion. Devices such as NexRing and the 6-in-1 Remote Health Monitor were chosen for their ability to track vital signs while maintaining user-friendliness, ensuring accessibility and ease of use for a wide range of individuals in everyday life. Finally, to meet specific monitoring needs that prioritize minimal disturbance to individuals, particularly in complex care scenarios, the XeThru X4M200 respiration sensor, a non-contact device, was included. By selecting sensors with diverse functionalities and designs, ranging from wearable devices to non-continuous and non-contact monitoring systems, this study tried to comprehensively address the real-world monitoring requirements of vulnerable populations.

The comparative analysis highlighted the diverse capabilities and applications of each device for health monitoring. EmotiBit emerged as a highly versatile tool, capable of providing comprehensive physiological data across multiple body locations. However, its setup and operation may require technical expertise, posing challenges for users with minimal experience in technology or programming. Similarly, the XeThru X4M200 respiration sensor offers different respiratory profiles to better adapt to specific use cases and supports non-contact respiratory monitoring through obstacles. Despite these innovative features, its signals can be difficult to interpret for users without a technical background, and its sensitivity to environmental factors, such as vibrations, along with the need for the person to remain still during breathing data capture, reduces its practicality in dynamic settings.

OpenGo Sensor Insoles and NexRing represent valuable wearable options for health monitoring, each with unique strengths. OpenGo Sensor Insoles offer multiple data recording modes, enabling monitoring tailored to specific needs. However, their functionality is limited to motion and activity-related metrics, requiring integration with other devices for a more comprehensive assessment of human health. NexRing, on the other hand, features user-friendly interfaces and supports the continuous tracking of vital signs and activity, making it suitable for daily health monitoring and sleep analysis. Its long battery life is a significant advantage, though its periodic updates (e.g., HR data every five minutes) limit its use in critical scenarios that demand real-time monitoring. The 6-in-1 Remote Health Monitor is able to track multiple vital parameters with minimal invasiveness and provides intuitive interfaces to manage recordings and view health data. However, its non-continuous nature and dependence on user interaction limit its effectiveness for ongoing monitoring.

Among the wearable devices analyzed, OpenGo Sensor Insoles and NexRing stand out for their discreet designs, allowing easy integration with clothing while minimizing interference with users’ daily routines. In contrast, EmotiBit features a less discreet design, making it less compatible with everyday attire. However, EmotiBit offers significant flexibility as it can be worn in various locations on the body, unlike the insoles or the ring, which are limited to fixed positions. However, although EmotiBit’s versatility in placement can address specific monitoring needs, it does not necessarily enhance its usability in everyday contexts. Optimal sensor placements, such as the fingertip or forehead for capturing high-quality PPG and EDA signals [78,84], are ideal for research or clinical settings but are often impractical for daily use. To promote the adoption of the device in everyday life, more conventional positioning options (e.g., the wrist [78]) should be considered. In such cases, signal processing techniques to filter out noise from suboptimal positioning become crucial to ensuring accurate measurements.

Another critical consideration arising from the comparison is the diverse availability of raw data. Not all devices provide direct access to unprocessed data, which is essential for advanced signal processing and the calculation of specific parameters. Many commercial devices instead offer pre-processed data, such as averages or trends, presented in simplified reports for immediate use. While these reports enhance usability and ease interpretation, they limit the device’s utility in scientific research or applications requiring detailed health monitoring. Based on this distinction, the analyzed sensors can be divided into two main categories. Consumer-grade devices, such as NexRing and the 6-in-1 Remote Health Monitor, prioritize user-friendliness by offering data already processed. This approach minimizes the risk of misinterpretation by non-expert users but restricts access to raw data, reducing the potential for advanced analysis. On the other hand, research-grade devices, including EmotiBit, OpenGo Sensor Insoles, and XeThru X4M200, provide direct access to raw data, offering higher levels of detail and supporting customized analysis methodologies, making them ideal for research and specialized monitoring needs. All this information is essential for making informed decisions about which sensor would deliver optimal performance and reliability across diverse healthcare contexts.

### 5.2. Suggestions for Sensor Selection

The selection of the most suitable sensor depends heavily on the intended healthcare application. Consumer-grade devices may be appropriate for autonomous or continuous monitoring in scenarios where device interaction needs to be simple, and real-time monitoring is not strictly critical. For instance, such devices could be suitable for managing chronic conditions in self-sufficient individuals who do not require intensive medical oversight. Subjects with hypertension could benefit from the use of a consumer-grade device such as the 6-in-1 Remote Health Monitor for BP tracking, in order to gather insights into their cardiovascular health and share data with healthcare professionals during periodic check-ups [100]. Another use case for consumer-grade devices could be the general fitness monitoring of older adults who are generally healthy but aim to maintain an active lifestyle [101]. Elderly individuals using NexRing could monitor their daily physical activity by tracking steps, calories burned, and HR, to ensure consistent levels of activity while avoiding overexertion.

However, consumer-grade devices might not be adequate in situations requiring advanced health monitoring or in cases of complex pathologies, especially in non-autonomous individuals or those with comorbidities prone to rapid progression or frequent relapses. In such cases, the need for more precise data and personalized analysis becomes crucial, and research-grade devices that offer direct access to raw data and greater control over measurements can be highly beneficial. For example, non-autonomous individuals with multiple chronic conditions, such as diabetes, hypertension, and chronic obstructive pulmonary disease, could benefit from advanced monitoring with devices such as EmotiBit. This tool could provide detailed data on HR, RR, and skin temperature, offering a comprehensive view of the health status and enabling caregivers to identify early signs of complications. Additionally, devices such as OpenGo Sensor Insoles could provide an in-depth analysis of gait dysfunctions in stroke survivors, helping to prevent falls, assess progresses during physical activity interventions, and enhance the recovery of motor functions [102]. Similarly, the XeThru X4M200 respiration sensor could be particularly useful for a detailed monitoring of sleep patterns and respiratory disturbances in people with Alzheimer’s disease, facilitating better management of related issues [103,104]. In some cases, a combination of consumer-grade and research-grade devices may be advantageous. A person could use a research-grade device during critical post-surgery weeks and transition to a consumer-grade wearable for long-term monitoring. Alternatively, a hybrid approach could involve both types of devices, ensuring continuous user engagement while providing intermittent, high-precision health insights. Therefore, the choice of the appropriate device for health monitoring primarily depends on the specific monitoring context and individual’s needs.

The selection of the device should also take into account other crucial factors that could influence its adoption and effective use in daily life. Beyond the monitoring context and the person’s specific health condition, it is essential to consider the sensor’s battery life and the user’s inclination to engage with the device. Some individuals might feel discomfort or embarrassment if the device does not integrate discreetly with their clothing or lifestyle. Others, particularly those with cognitive impairments, may unintentionally remove the device, potentially disrupting monitoring and putting their health at risk. Additionally, the user’s level of technological literacy is a key consideration. Individuals unfamiliar with technology might face challenges in using the device or its associated platforms, leading to frustration or abandonment. To address this concern, comprehensive training and support programs should be implemented, with the aim to not only teach users how to operate the devices but also emphasize the benefits they provide for daily health management. A specific focus should also be given to explaining the meaning of the measurements collected by each device, in order to ensure people understand their relevance and avoid unnecessary concern.

### 5.3. Wearability and Comfort Across Different User Groups

Wearability and comfort are essential for health monitoring sensors to ensure a positive user experience. Meeting the diverse needs of groups such as the elderly, children, and athletes requires personalized designs that balance comfort, accessibility, and functionality. For elderly users, for example, usability and accessibility are critical. Devices tailored to this group often incorporate clear and easily readable displays, as well as user-friendly wearable formats such as wristbands [105]. Non-invasive or minimally invasive options, including on-skin patches or non-contact sensors, offer significant advantages for individuals with sensitive skin or limited mobility, enabling seamless integration into daily routines while minimizing discomfort [29,106]. For children, sensor designs emphasize durability and safety to accommodate their higher activity levels and sensitivity to discomfort. These devices typically incorporate lightweight, flexible materials and smaller sizes to enhance comfort, while using safe construction materials and playful aesthetics to encourage consistent use without compromising functionality [107]. In contrast, athletes require health monitoring sensors specifically designed to endure intense physical activity. For this group, designs focus on flexibility, sweat resistance, and ergonomic placement, ensuring that sensors remain durable and provide accurate data even during high-intensity movements. These attributes facilitate reliable monitoring without impeding performance or comfort during rigorous training and competition [108].

The sensors discussed in this analysis offer various solutions to address the unique needs of different user groups. EmotiBit, for example, features adjustable straps that make it highly versatile for users of all ages, from children to elderly individuals. Its customizable case further enhances usability during dynamic activities, making it a suitable option for athletes. Alternatively, OpenGo Sensor Insoles and NexRing adopt a design approach focused on seamless integration with daily clothing, making them particularly appealing for elderly users who prioritize comfort and ease of use. Devices such as the XeThru X4M200 respiration sensor are suitable for older adults and children who benefit from minimal interference with daily routines. Also, the 6-in-1 Remote Health Monitor, with its intuitive design, is a strong candidate for elderly users, although those with limited hand mobility may require assistance with certain features.

By providing a range of options suitable for the specific needs of diverse user groups, these devices hold significant potential for widespread adoption in health monitoring. Moreover, an early user engagement can help identify key comfort and usability requirements, ensuring that the devices remain effective, accessible, and appealing to all users.

### 5.4. Challenges in Sensor Performance in Dynamic Environments

Health monitoring sensors are increasingly required to operate effectively in complex and dynamic environments, including outdoor settings, hospitals, and homes, where factors such as temperature fluctuations, humidity, physical movement, and electromagnetic interference can significantly influence their performance. Maintaining stability and accuracy over extended periods in these conditions is a considerable challenge and, to mitigate these environmental impacts, current health monitoring sensors are equipped with a variety of advanced features. Many wearable sensors incorporate signal processing algorithms that filter noise and reduce motion artifacts, ensuring precise data collection even in high-activity or outdoor settings [109]. Non-contact sensors, which use technologies like radar or imaging, are less affected by environmental variables such as lighting or physical barriers [37,38,39]. Additionally, many devices include temperature and humidity compensation mechanisms, helping them maintain accuracy and stability in environments with variable conditions. For instance, devices such as OpenGo Sensor Insoles and NexRing are optimized for both indoor and outdoor use, with the ability to endure a wide temperature range and varying humidity levels, ensuring stability in different settings. Similarly, sensors like EmotiBit are designed to withstand a variety of indoor and outdoor environments, with features that make it heat and light-resistant, although it is not waterproof.

Despite the advances in sensor technology, there are still opportunities for improvement. For example, integrating adaptive machine learning models could enable real-time self-calibration, allowing sensors to better respond to changing environmental conditions [110]. Furthermore, enhancing material durability and waterproofing would boost sensor performance in outdoor settings [111]. Future sensor designs might also focus on multi-sensor integration, combining data from various modalities to provide more comprehensive and reliable measurements in diverse environments [6].

### 5.5. Security, Privacy, and Ethical Issues in Sensors for Health Monitoring

The integration of sensors into healthcare applications brings significant benefits but also raises critical ethical concerns, particularly regarding data privacy and security. These issues are pivotal in the selection of monitoring devices, as the ability to safeguard sensitive health data is a key determinant of a sensor’s suitability for clinical use.

One major challenge in health monitoring devices is ensuring the secure collection, processing, and storage of data. Current devices employ several measures to protect privacy and security. These include the use of end-to-end encryption to secure data during transmission between devices and connected platforms, as well as access control mechanisms such as password-protected accounts, biometric authentication, or two-factor authentication to prevent unauthorized access. Additionally, many devices use anonymization or pseudonymization techniques to protect users’ identities during data analysis. Compliance with privacy and security regulations, such as the GDPR, further ensures these devices meet rigorous standards for safeguarding sensitive health information [112,113,114].

To further enhance data privacy and security in health monitoring devices, several advancements can be implemented. These include adopting more advanced encryption protocols, such as quantum-resistant algorithms, to future-proof data security and utilizing edge computing to process data locally on devices, thereby reducing the vulnerabilities associated with cloud storage. Empowering users with granular control over data permissions would allow them to decide what information is shared and with whom, enhancing transparency and trust [115,116]. Regular security audits and updates are essential to address emerging threats, ensuring that devices remain resilient against evolving cybersecurity challenges. The potential integration of blockchain technology presents another promising avenue; it could facilitate secure, decentralized data management while allowing users greater control over their personal health information [117]. Artificial intelligence (AI) could also play a significant role in future developments by enabling real-time threat detection systems that monitor for unusual access patterns or potential breaches [118].

Another ethical consideration is obtaining informed consent from users. It is crucial to ensure that people fully understand how their data will be used, stored, and shared, particularly in cloud-based or AI systems. Transparent communication on these aspects is essential to maintain trust and encourage adoption [119].

By addressing these issues, the healthcare sector can ensure that sensor technologies align with the principles of data privacy and security. Many of these considerations, including anonymization techniques, user-controlled data sharing, and adaptive consent, are already being incorporated into the design of current sensor technologies and will continue to improve the ethical integrity and user acceptance.

### 5.6. Study Limitations and Future Directions

Some limitations may arise from this analysis. First, the selection of devices, particularly for the non-continuous and non-contact categories, was not exhaustive. Although the selected branded products may not fully capture the diversity within their respective categories, they were chosen to provide clear examples that highlight the characteristics and applications of each sensor type. Nevertheless, other devices with comparable or superior capabilities may exist and offer additional insights. Future research should consider including a broader range of devices within each sensor category to enhance the robustness of the findings.

Additionally, future authors should expand the framework by incorporating newer sensor technologies, such as hybrid or AI-enabled sensors, ensuring the study remains relevant as the field evolves. Recent advances in hybrid sensors enable the simultaneous recording of multiple biophysical and biochemical signals on a single platform [120]. For example, combining electromyogram signal detection with sweat cortisol monitoring offers a multi-dimensional approach to stress assessment and early detection of abnormal physiological changes [121]. Furthermore, progress in flexible hybrid electronics has led to skin-conformable sensors that enhance compliance with the human body [122]. Moreover, AI integration in these sensors enables real-time health monitoring by analyzing data efficiently, identifying complex patterns, and providing predictive insights, making them powerful tools for future healthcare applications [123].

Another limitation arising from our study is that our classification of sensors into wearable, non-continuous, and non-contact categories does not fully account for the overlapping functionalities of these devices. Many sensors feature characteristics that span multiple categories, making strict classifications challenging. For instance, a wearable sensor might also function as non-continuous if used periodically, thus providing intermittent data while maintaining its wearable design. Similarly, a non-continuous sensor may also be considered non-contact if it operates without direct skin contact, such as temperature measurement devices. In our approach, we classified sensors based on a comprehensive evaluation of their functionality, usability, and design. This categorization was intended to offer an intuitive and practical framework for our target audience, in line with the existing literature, while recognizing that many devices integrate multiple functionalities.

Finally, the lack of direct testing among selected devices limits our findings in real-world performance. The primary goal of this analysis was to provide an initial comparison of existing sensors, which could potentially guide the selection of the most suitable technology for specific healthcare scenarios. Future research should focus on studies that evaluate sensor performance in real-world settings, including diverse user populations and environmental conditions. The impact of factors such as user compliance, device wearability, and data interpretation should also be assessed in these studies.

## 6. Conclusions

Our comparative analysis underscores the significant potential of wearable, non-continuous monitoring, and non-contact sensors in transforming health monitoring practices. Each sensor type presents unique strengths and limitations, making it essential for healthcare stakeholders to carefully consider the specific health conditions and monitoring requirements of individuals when selecting appropriate technologies.

The findings indicate that, while these devices can facilitate proactive healthcare by enabling ongoing data collection and real-time monitoring, their successful integration into everyday life hinges on several critical factors. These include user comfort, ease of use, technological proficiency, and the ability to adapt the technology to meet individual health needs. Moreover, the economic accessibility of these devices plays a crucial role in their adoption, particularly in resource-limited settings.

Despite the promising capabilities of the selected sensors, the study acknowledges certain limitations, such as the non-exhaustive nature of the device selection and the lack of direct performance testing in real-world scenarios. Future research should evaluate these technologies across diverse users and environments to clarify their practical implications. It should also investigate the influence of user compliance and data interpretation methods on health outcomes.

In conclusion, the integration of user-centered sensor technologies into healthcare systems has the potential to significantly improve quality of care and promote better health. By fostering a deeper understanding of the strengths and weaknesses of different sensor types, this analysis serves as a valuable resource for researchers, healthcare providers, and policymakers, ultimately contributing to the advancement of personalized healthcare solutions.

## Figures and Tables

**Table 1:** Comparison of selected sensors in measuring essential health parameters.

Category	EmotiBit	OpenGo Sensor Insoles	NexRing	6-in-1 Remote Health Monitor	XeThru X4M200 Respiration Sensor
**HR**	✓		✓	✓	✓
**HRV**	✓		✓ *	✓	✓
**RR**	✓		✓ *	✓	✓
**SpO2**	✓		✓ *	✓	
**BP**	✓			✓	
**Body Temperature**	✓		✓ *	✓	
**Sleep**	✓		✓		✓
**Activity**	✓	✓	✓		✓

* Measured only during sleep. HR: heart rate; HRV: heart rate variability; RR: respiratory rate; SpO2: blood oxygen saturation; BP: blood pressure.

**Table 2:** Comparison of selected sensors based on recording modes and sensor functionality.

Category	EmotiBit	OpenGo Sensor Insoles	NexRing	6-in-1 Remote Health Monitor	XeThru X4M200 Respiration Sensor
Recording Modes and Sampling Rates	Continuous recording, manual initiation; sampling rates: 25 or 100 Hz (PPG), 15 Hz (EDA), 7.5 Hz (temperature), 25 Hz (IMU)	Continuous recording, manual initiation; configurable sampling rates (10, 25, 50, or 100 Hz) and sensor channel; preset recording durations	Continuous recording, automatic initiation; workout and mindfulness sessions with predefined durations	Non-continuous recording, manual initiation; sampling rate for ECG: 512 Hz	Continuous recording, manual initiation; sampling rate: 23.328 GS/s
Memory and Data Storage Capacity	High-speed microSD card, 32 GB	32 MB onboard memory, up to 32 h of recording	1-week data storage	N/A (data stored in app)	N/A (internal buffer)
Calibration and Initialization Requirements	Factory-calibrated, 5-minute warm-up recommended	Factory-calibrated; initial weight calibration, warm-up required	Factory-calibrated	Calibration recommended every 2 years	Factory-calibrated
Sensor Sensitivity	Affected by body movement, fit, and body position	Affected by fit and external interference (Wi-Fi, Bluetooth)	Affected by fit	Affected by incorrect positioning, movement, and subject’s conditions	Affected by vibrations, indirect reflections, material properties
Environmental Suitability	Indoor/outdoor; heat/light resistant, non-waterproof	Indoor/outdoor; temperature range: −10 °C to +50 °C, humidity: 5% to 95%	Indoor/outdoor; durable for extreme conditions (hot tubs, saunas, cryotherapy)	Mainly indoor use; sensitive to environmental factors; temperature range: 5 °C to 40 °C, humidity: 15% to 93%	Indoor/outdoor; operates in extreme temperatures (−40 °C to +85 °C), sensitive to moisture

**Table 3:** Comparison of selected sensors based on comfort, design, and usability.

Category	EmotiBit	OpenGo Sensor Insoles	NexRing	6-in-1 Remote Health Monitor	XeThru X4M200 Respiration Sensor
Wearability, Placement, and Comfort	Worn in any orientation and almost anywhere on the body with stretchable straps; less discreet, more intrusive	Worn inside closed shoes; slim and flexible; minimal invasiveness; initial mild discomfort for some users	Worn on the index finger; minimal invasiveness; comfortable for prolonged used	Requires specific positioning based on measurements; minimal invasiveness	Non-contact; positioned on desks, walls, or ceilings; requires stable surfaces for optimal recording
Dimensions, Portability, and Aesthetics	Portable; length: 6.1 cm; width: 2.7 cm; thickness: 2.0 cm; weight: 20–25 g; may not blend seamlessly with clothing	Portable; length: 21.5–31.8 cm; width: 8.0–11.0 cm; thickness: 1.1 cm; weight: 63–102 g; compatible with most footwear	Portable; diameter: 1.8–2.3 cm; circumference: 0.6–7.1 cm; width: 0.8 cm; thickness: 0.3 cm; weight: 4–6 g; in four colors; compatible with everyday clothing	Portable; length: 7.0 cm; width: 7.0 cm; thickness: 1.8 cm; weight: 70 g; compact and easy to carry in a bag for use anywhere	Portable; length: 5.8 cm; width: 3.7 cm; integrates well into home environments
Physical Constraints	Compatible with various body sizes; adjustable straps	Nine double sizes; not suitable for custom orthotics or users over 120 kg	Seven sizes for different finger dimensions	Circumference of arm cuff: 22–35 cm; some difficulty for users with limited mobility	No physical constraints
Reusability and Cleaning	Reusable; cleanable with alcohol wipes; removable and cleanable electrodes; hygiene covers	Reusable; cleaned with disinfectants and a damp soft wipe	Reusable after cleaning and app data reset; cleaned weekly with soft cloth or mild soap and water	Reusable; clean with ethanol or neutral agents; not waterproof	Reusable; minimal cleaning required due to non-contact nature

**Table 4:** Comparison of selected sensors based on sensor platforms and support resources.

Category	EmotiBit	OpenGo Sensor Insoles	NexRing	6-in-1 Remote Health Monitor	XeThru X4M200 Respiration Sensor
Platform Compatibility and Functionality	Open-source software for signal visualization, recording, streaming, and parsing; compatible with Windows (10+), macOS, and Linux	Mobile app for data recording and transfer; compatible with Android 9 (Pie) and Bluetooth Low Energy 5.0; software for receiving, analyzing, and exporting data; compatible with Windows (10+)	Mobile app for real-time tracking and trend monitoring; compatible with Android (6+), iOS (13+), and Bluetooth Low Energy 5.1	Mobile app for data recording and historical trends; compatible with Android (5+), iOS (11+), and Bluetooth 4.0	Software for configuring, visualizing, and managing data output; compatible with Windows (7/10), Ubuntu, and OSX
Usability and Accessibility of Platforms Interfaces	Moderate usability; intuitive interfaces for experts; requires technical expertise for assembly and operation	Intuitive interfaces for app and software; software with customizable dashboards	Intuitive and detailed app interfaces	Intuitive and simple app interfaces	Moderate usability; intuitive interfaces for experts
Operational Indicators and Alerts	LEDs for status (e.g., Wi-Fi connection, low battery, recording); battery level displayed in the software	Charging LEDs, app indicators, zeroing status, pre-measurement pop-ups, sensor warnings, biofeedback alerts	App notifications for low battery and connectivity; charging dock LED indicator	LEDs and vibration for power/charging; limited app prompts for incorrect setup	LEDs for status; real-time color cues in software for activity detection
Support and Documentation Quality	Detailed documentation, FAQs, Python and MATLAB libraries, community forum	Extensive resources (manuals, videos, FAQs); Python library and programmer’s guide	Comprehensive in-app instructions, brief user manual, online video tutorials	Detailed user manual, online video tutorials, FAQs, basic troubleshooting guidance for errors	Comprehensive online documentation and user guide
Developer Tool Availability	Customizable firmware; integration with external platforms	Python library and SDKs for endpoint, insole, and mobile app development	iOS/Android SDK for custom endpoint solutions	SDK for integration with external platforms	Development kit and API with MATLAB, Python, and C++ support for extensive customization

**Table 5:** Comparison of selected sensors based on data transfer, accessibility, and export features.

Category	EmotiBit	OpenGo Sensor Insoles	NexRing	6-in-1 Remote Health Monitor	XeThru X4M200 Respiration Sensor
Data Transfer	Wi-Fi to software; automatic transfer	Automatic transfer via Bluetooth to mobile app; automatic or manual transfer via Wi-Fi to desktop software	Bluetooth to app; automatic transfer	Bluetooth to app; automatic transfer	Micro USB cable to PC; automatic transfer
Data Accessibility	Data accessible after recording, even if disconnected	Data accessible after recording, even if disconnected	Data viewed exclusively via the mobile app, even if disconnected	Data accessible after recording, even if disconnected	Data accessible after recording, even if disconnected
Raw Data Export	.csv for raw data stored on the onboard SD card; .csv for parsed files	.txt for raw data; easily exported from the software	Not supported	Not supported	.dat for raw data, automatically stored in a user-specified directory
Reporting Features	Not supported	Different types of automated reports, in .pdf and .xlsx formats	Not supported	PDF reports for single measures; limited graphical content	Not supported

**Table 6:** Comparison of selected sensors based on battery performance and power management.

Category	EmotiBit	OpenGo Sensor Insoles	NexRing	6-in-1 Remote Health Monitor	XeThru X4M200 Respiration Sensor
Battery Life	2.5–4 h (live streaming), 8–9 h (recording with Wi-Fi off)	8–15 h (continuous recording)	4–7 days	500 charge cycles (not designed for continuous monitoring)	N/A (requires external power)
Charging Method	Micro USB charging	Coin cell (PD2032), charging slot with micro USB cable	Wireless charging (USB charging dock)	Micro USB charging	Micro USB or 16-pin connector
Recharge Time	2 h	2.5 h	10 min–1.5 h	3–4 h	N/A (requires external power)
Power-Saving Features	Low Power Mode, Wi-Fi Off Mode, Sleep Mode, Hibernate switch	Sleep Mode, optimized sensor setup for battery conservation	Power Saving Mode	Low-performance mode for ECG measurements	N/A (requires external power)

**Table 7:** Comparison of selected sensors based on costs and economic considerations.

Category	EmotiBit	OpenGo Sensor Insoles	NexRing	6-in-1 Remote Health Monitor	XeThru X4M200 Respiration Sensor
Initial Price	EUR 462.95 (Bundle)	EUR 1895 (Sensor Insoles) + EUR 995 (Base Module)	USD 249.00	USD 249.00	Approximately USD 400
Subscription Requirements	No subscription required	No subscription required	No subscription required	No subscription required	No subscription required
Additional Costs	Battery/sensor replacements	SDK packages, customer support, software updates, sensor replacement, charging accessories	N/D	N/D	N/D

## Data Availability

The original contributions presented in the study are included in the article, further inquiries can be directed to the corresponding author.
